# An Assumed Vertical Transmission of SARS-CoV-2 During Pregnancy: A Case Report and Review of Literature

**DOI:** 10.7759/cureus.10659

**Published:** 2020-09-26

**Authors:** Nirmal K Mohakud, Hari Yerru, Monalisha Rajguru, Sushree S Naik

**Affiliations:** 1 Pediatric Medicine, Kalinga Institute of Medical Sciences, Bhubaneswar, IND; 2 Obstetrics and Gynecology, Kalinga Institute of Medical Sciences, Bhubaneswar, IND; 3 Obstetrics and Gynecology, Capital Hospital, Bhubaneswar, IND

**Keywords:** rt-pcr, pneumonia, covid-19, sars-cov-2, neonate, vertical transmission

## Abstract

The ongoing coronavirus disease 2019 (COVID-19) pandemic caused by severe acute respiratory syndrome coronavirus 2 (SARS-CoV-2) has affected persons of all ages, including the newborns. Few published case reports and case series have described the possibility of vertical transmission of COVID-19. In the present report, we describe a young primigravida at 33 weeks of gestation, who presented with a four-day history of low-grade fever, malaise, and breathing difficulty. She underwent testing of nasopharyngeal swab sample by real-time polymerase chain reaction (RT-PCR), which was positive for COVID-19. Cesarean section was done, and a preterm low birthweight baby was delivered. The baby required resuscitation at birth and was mechanically ventilated for a shorter duration. A tracheal aspirate that was taken at 12 hours of life tested positive for COVID-19. The course and outcome of the newborn are described here along with the possibility of vertical transmission.

## Introduction

The ongoing coronavirus disease 2019 (COVID-19) pandemic caused by severe acute respiratory syndrome coronavirus 2 (SARS-CoV-2) has affected persons of all ages, including the newborns [1]. Few published case reports and case series have described the newborns affected with COVID-19 [2,3]. In these publications, assumptions have been made (vertical vs horizontal) regarding the mode of transmission from the infected mothers. Although few publications have suggested a vertical (in utero) transmission, others have refuted this mechanism, and instead, emphasize a horizontal mode of transmission [4-8]. In the present report, we describe a premature newborn, who was born to a primigravida mother with HELLP (hemolysis, elevated liver enzymes, and low platelet count) syndrome and moderate COVID-19 pneumonia. The newborn tested positive at 12 hours of life for COVID-19 by real-time polymerase chain reaction (RT-PCR) of the tracheal aspirate sample [9]. It has to be noted that HELLP syndrome is a serious complication in pregnancy that occurs in 0.5%-0.9% of all pregnancies, and in 10%-20% of severe pre-eclampsia cases [10].

## Case presentation

A primigravida aged 30 years under quarantine complained of sore throat, low-grade fever, and malaise. She got tested and found positive for COVID-19. After two to three days, she developed breathing difficulty, decreased fetal movements, edema, and visual disturbance. Finally, she got referred to us for the subsequent management of her illness. She had already received two doses of TT (tetanus toxoid) injections and was on vitamin and mineral supplements (iron/folic acid and calcium tablets) regularly. She was also taking thyroxine tablet (25 µg) once daily for hypothyroidism. She developed hypertension in the second month of pregnancy. Ultrasound done at 33 weeks of gestation revealed a single, live fetus (weight = 930 g), and the amniotic fluid index (AFI) was 9. She was conscious at admission with the following vital parameters: temperature 98.6˚F, pulse rate 112/min, respiratory rate 30/min, and blood pressure 190/110 mmHg. General examination showed pallor, edema, and icterus. There was 50 ml of bloody urine in the urinary bag. She was finally diagnosed to have HELLP syndrome with hypothyroidism and moderate COVID-19 pneumonia (Table 1) [9].

**Table 1:** Laboratory results of the mother TLC: total leukocyte count; ALC: absolute lymphocyte count; CRP: C-reactive protein; CPK: creatine phosphokinase; AST: aspartate transaminase; ALT: alanine transaminase; ABG, arterial blood gas

Variables	Reference range	Results during the hospital stay			
		Day 0 (admission)	Day 1 (24 hours)	Day 2	Day 5
Hemoglobin (g/dL)	12–15	8.6	8.9	8.9	9.3
TLC (/cumm )	4,000–10,000	12,640	10,400	8,800	8,700
Platelet count (/cumm)	1.5–4.5 lakh	0.5	0.18	0.25	0.86
ALC (/cumm)	1,000–3,000	2,022	1,894	1,900	2,430
CRP (mg/dL)	<5.0	12.0	<5.0	<5.0	-
Procalcitonin ng/mL	<0.5	0.51	-	-	-
D-dimer (µg/mL)	<0.5	5.7	3.2	<0.5	-
Ferritin (ng/mL)	12–150	630	428	256	-
CPK (U/L)	<170	52	-	-	-
Random blood glucose (mg/dL)	70–140	92	1,111	102	-
Creatinine (mg/dL)	0.5–1.04	1.03	0.9	0.7	-
Urea (mg/dL)	7–17	41	32	30	-
Total bilirubin (mg/dL)	0.2–1.20	7.8	7.1	6.8	5.0
AST (U/L)	14–36	220	176	165	132
ALT (U/L)	9–52	164	158	120	98
Sodium (mEq/L)	135–145	130	134	131	132
Potassium (mEq/L)	3.5–5.1	4.8	4.2	3.5	3.6
Chloride (mEq/L)	98–107	100	101	104	104
ABG		Respiratory alkalosis	Respiratory alkalosis	Normal	Normal
Chest X-ray		Bilateral heterogeneous opacities	-	-	Normal

**Figure 1:**
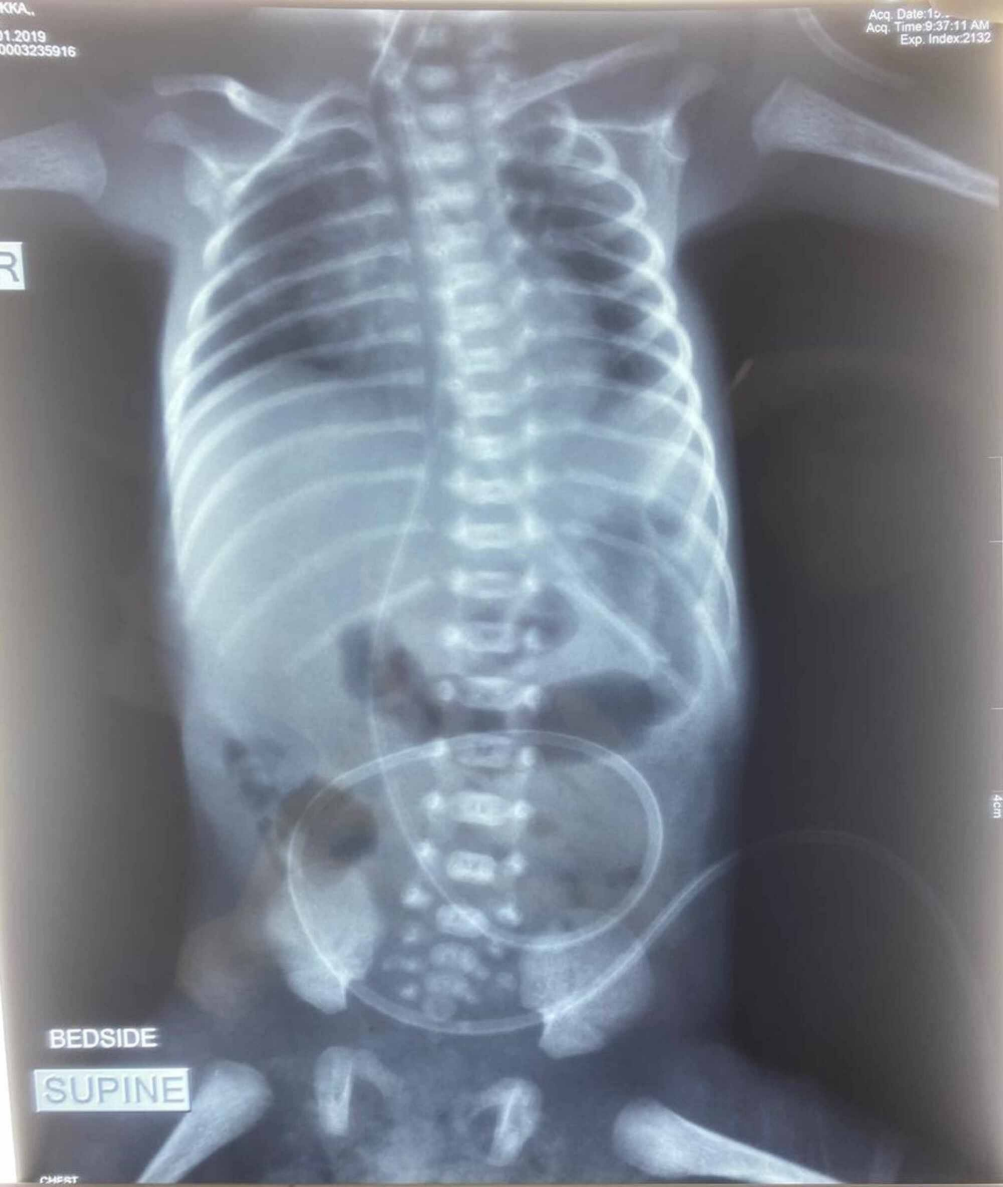
Chest X-ray of the newborn done at one hour of age X-ray chest anteroposterior view with ventilator in situ showing bilateral good air entry.

Management was done as per the hospital policy and Government guidelines on COVID-19. She underwent a cesarean section, and a male newborn (weight = 930 g) was delivered. The newborn did not cry immediately (Apgar at 1 min 3/10, after 5 min 5/10), and required resuscitation. Mechanical ventilation (synchronized intermittent mandatory ventilation [SIMV] mode, FiO_2_ 25%, peak inspiratory pressure/positive end-expiratory pressure [PIP/PEEP] 12/5 cm of H_2_O, at a rate of 30/min) was started without delayed cord clamping and skin-to-skin contact. A chest X-ray done was normal (Figure 1) [9].

The newborn threw a seizure after two hours for which relevant investigations were done (Table 2) [9].

**Table 2:** Laboratory results of the newborn TLC: total leukocyte count; ALC: absolute lymphocyte count; CRP: C-reactive protein; CPK: creatine phosphokinase; AST: aspartate transaminase; ALT: alanine transaminase

Variables	Reference range	Results during the hospital stay		
		Day 0 (admission)	Day 1 (24 hours)	Day 2
Hemoglobin (g/dL)	15–21	17.0	18.0	16.5
TLC (/cumm )	5,000–10,000	2,800	5,400	10,200
Platelet count (/cumm)	1.5–4.5 lakh	1.59	1.80	1.78
ALC (/cumm)	1,000–3,000	1,008	1,942	3,668
CRP (mg/dL)	<5	3	-	-
Total calcium (mg/dL)	7.2–11.0	8.8	-	-
CPK (U/L)	<170	210	148	-
Random blood glucose (mg/dL)	>40	62	124	-
Creatinine (mg/dL)	0.5–1.04	1.1	1.0	1.0
Urea (mg/dL)	12–42	44	38	36
Total bilirubin (mg/dL)	<5	4.2	4.1	4.1
AST (U/L)	47–150	170	148	134
ALT (U/L)	13–45	52	44	44
Sodium (mEq/L)	135–145	132	135	137
Potassium (mEq/L)	3.5–5.1	5.1	4.9	4.6
Chloride (mEq/L)	98–107	104	101	105
pH	7.35–7.43	7.19	7.32	7.34
Chest X-ray	Normal	Normal	-	-

Injection phenobarbitone was started along with intravenous antibiotics (cefotaxime plus amikacin). There was no further recurrence of seizure. As the condition of the newborn improved, he was extubated to continuous positive airway pressure (CPAP) after 24 hours of mechanical ventilation. RT-PCR of the tracheal aspirate sample that was taken after 12 hours found positive for COVID-19. However, he showed consistent improvement, was weaned off oxygen, and discharged in a healthy condition.

## Discussion

While reviewing the literature, we could find a few published case reports and case series on neonatal SARS-CoV-2 (COVID-19) infection. The authors in one review reported 179 cases of newborns tested positive at birth, whose mothers were infected in the third trimester of pregnancy [5]. The time interval (mean, between delivery, and infection) was three days (range, 0-25). The RT-PCR was negative when performed on cord blood and amniotic fluid. However, COVID-19 was tested positive in the nasopharyngeal samples of only six newborns: 1 (at 16 hours of life), 2 (at 36 hours of life), and 3 (at 48 hours of life). The authors kept following possibilities in their review of these cases: contact with infected parents or healthcare professionals (transmission at birth) or via breastfeeding, and droplet inhalation. However, these newborns were born through cesarean delivery, separated immediately from their mothers, and instead placed in another room for isolation. In addition, It has to be remembered that breast milk does not transmit COVID-19 [11]. Therefore, vertical transmission (transplacental/in utero) remains a possibility, which is difficult to rule out [5]. Similarly, in the index case, the mother was possibly infected in the third trimester and underwent a cesarean section. The newborn was transferred to the neonatal ICU without contact with his mother.

To date, there is a lack of clarity regarding the criteria of congenital COVID-19 infection. The authors of one study described that three newborns born to mothers with COVID-19 infection had positive antibodies (IgM and IgG) at birth [7,8]. Whether they need to be labeled as congenital infections (vertical transmission) is debated (because IgM antibodies do not cross the placenta) [5]. In a case series including 33 newborns, 3 tested positive for COVID-19 by RT-PCR of nasopharyngeal and anal swab samples on day 2 and day 4 of life. They developed severe pneumonia [8]. The authors were not sure about the timing of transmission (perinatal or postnatal), as they were born by cesarean section, like the index case, and were immediately separated from the mothers. In the present report, the index newborn was tested positive at 12 hours of life without any features of symptomatic COVID-19 infection [9]. Published studies have shown that maternal COVID-19 infection can cause premature delivery, respiratory distress, and intrauterine fetal death [3,5,12]. These complications result from severe hypoxemia as a result of pneumonia in the mother. In the present report, decreased fetal movement and perinatal asphyxia could be explained by these factors as the mother had moderate COVID-19 pneumonia with HELLP syndrome.

The present report has some limitations. Due to lack of logistics, the presence of the COVID-19 virus was not confirmed in body fluids (amniotic fluid, cord blood) or tissue specimens (placental tissue) that might have further clarified the mode of transmission. In addition, COVID-19 antibody testing also could not be performed in the newborn.

## Conclusions

Our report shows that COVID-19 infection may possibly get transmitted vertically to the fetus. More research is needed to better elucidate the mechanisms involved in this process.
